# Effect of lameness on feeding behavior of zero grazed Jersey dairy cows

**DOI:** 10.3389/fvets.2022.980238

**Published:** 2022-09-20

**Authors:** Sandra Gündel, Christian Looft, Leslie Foldager, Peter T. Thomsen

**Affiliations:** ^1^Department of Animal Breeding and Husbandry, Hochschule Neubrandenburg—University of Applied Sciences, Neubrandenburg, Germany; ^2^Department of Animal Science, Aarhus University, Aarhus, Denmark; ^3^Bioinformatics Research Centre, Aarhus University, Aarhus, Denmark

**Keywords:** feeding behavior, dairy cattle, Jersey cows, lameness, breed difference

## Abstract

The dairy industry faces major challenges with high levels of lameness, in parallel to an increased consumer focus on animal welfare. This encourages farmers to consider more robust breeds, such as Jersey cows. As little is known about the behavior of this breed under loose housing conditions, the present study sought to describe the feeding behavior of lame and non-lame Jersey cows in different parities. Such breed-specific information of behavioral changes is needed for breed-specific herd management decisions and may contribute to identifying animals that are susceptible to developing lameness in the future, thus reducing impacts on the welfare and production of cows. Feeding data from 116 Danish Jersey cows were collected using automatic feeders, and lameness status was assessed by technicians every second week. The cows were kept in a loose housing system, with cubicles, a slatted concrete floor, and automatic milking robots. Eating time per visit and per day, the number of visits per day, and intervals between meals were analyzed using generalized linear mixed effects models. The effect of lameness was not significant for any variable. Primiparous Jersey cows had significantly longer eating times per day, shorter meal intervals, and a lower number of visits per day than older Jersey cows. Week in lactation affected the eating time per visit and per day, the number of visits, and between-meal intervals. In conclusion, we found no differences between lame and non-lame Jersey cows but between parities, which disagree with previous research on other breeds, suggesting that Jersey cows not just differ in size and looks but also in their behavioral reaction when lame. Although data from only one herd of a research center were used, this study has demonstrated the need for further research about breed-specific differences and their implications for the health and welfare of the animals.

## Introduction

Achieving good animal welfare of high-producing dairy cows in intensively managed systems remains a major challenge for the dairy industry while facing consumer demands and changing climate conditions (1, 2). One of the most important welfare problems in dairy cows is lameness, as it causes pain and thus may reduce animal welfare and productivity (3, 4).

Risk factors associated with lameness range from housing environment and herd management, to genetic factors and breed (4). Compared to purebred Holstein cows, the smaller framed Jersey breed is receiving increasing interest from dairy farmers and scientists (5–7). The Jersey breed differs from Holstein cows not only in size and looks but it also produces milk with higher nutrient density and has a higher reproductive performance and heat tolerance (8, 9). Even though mainly used for grazing systems (10) and crossbreeding, pure-bred Jersey cows are currently, in numbers, the second largest dairy breed in many countries (11, 12). Recently, it has been demonstrated that Jersey cows have a lower carbon footprint per kg of fat-and-protein corrected milk produced compared to Holstein cows (13).

Nevertheless, knowledge remains limited about the impact of lameness on the feeding behavior of zero-grazed Jersey cows over the entire period of lactation. Knowledge about the effects of lameness on feeding behavior is important as the feeding behavior has a significant impact on productivity, and it may improve the early detection of lameness and thus have the potential to reduce impacts on the well-being and performance of cows (14, 15).

At present, there are indications that changes in eating time may indicate changes to the health status of a cow (14, 16), and connections between dairy cow behavior and lameness have been studied (17, 18). Grimm et al. (19) showed that lame cows eat fewer and shorter meals and have a lower intake per meal. So, the feeding behavior could be a predictive tool for the early detection of lame cows. Yet, despite the growing numbers of housed Jersey cows in Europe (5), most studies about the effect of lameness on feeding behavior focus on Holstein cows or grazed Jersey cows.

Thus, here, we aimed to describe and compare the feeding behavior of lame and non-lame Jersey cows in a loose housing system. We hypothesized that the behavior of lame Jersey cows would be affected in a similar way as has been described for lame Holstein cows, with lame cows having shorter and fewer visits to the feeder. Additionally, we expected primiparous cows to be having more visits of shorter duration and, consequently, shorter eating time per day and between-meal intervals.

## Materials and methods

### Animals

Data from 116 individual Danish Jersey cows housed at the Danish Cattle Research Center (Foulum, Denmark) were collected between 4 January, 2018 and 30 April, 2019. Because feed composition affects the feeding behavior of cows (20, 21), and to keep environmental conditions as constant as possible, only animals fed the standard partially mixed ration (PMR) were included. The proportion of cows within first and later parities was 40 and 60%, respectively. Parity ranged from one to eight lactations. The group composition was dynamic, with cows entering and leaving the experiment, depending on their expected calving dates. Unless moved to a hospital pen, cows that received veterinary treatment during lactation were not excluded from the study. Over the whole study period, 158 treatments have been counted. Reasons for handling spanned from mastitis over heat induction to routine hoof trimming. Ethical approval for the study was not needed according to the European and Danish regulations and current guidelines for the ethical use of animals in research.

### Housing and management

The cows were kept as one group in a loose housing system, with a slatted concrete floor and cubicles with mattresses (Comfi Cushion, Egtved, Denmark). The group of Jersey cows had free access to one automatic milking system (AMS) (DeLaval AB, Tumba, Sweden), water, and PMR, which was fed *ad libitum* using computerized feeding bins (Insentec Roughage Intake Control system; Insentec BV, Marknesse, Netherlands). Feed was delivered four times a day. Cows had access to 29 feed bins. The stocking density (animal to feed bin ratio) ranged from 1.8 to 2.3. Each feeder electronically identified individual cows, and cows were free to use any feeder.

**Figure 1:**
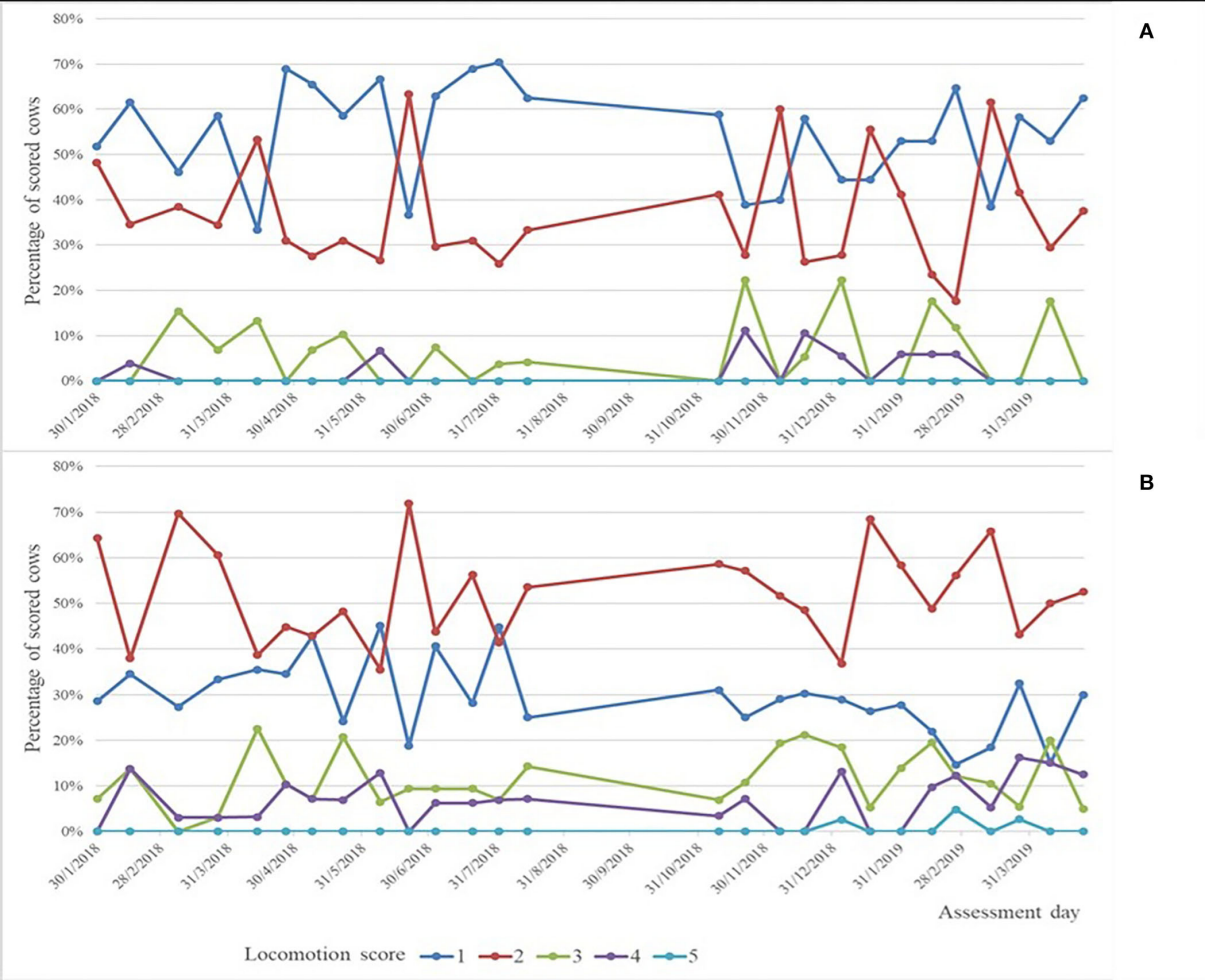
Percentage of cows assessed in each lameness score by assessment day for primiparous **(A)** and multiparous **(B)** cows.

Locomotion scoring (LS) of all cows was done while cows were walking freely along the aisle by experienced and calibrated technicians every second week using the scale described by Thomsen et al. (22) with LS1 = normal, LS2 = uneven gait, LS3 = mildly lame, LS4 = lame, and LS5 = severely lame. The distribution of scores by parity and assessment day is shown in Figure 1.

### Feeding behavior

All cows were allowed to feed on PMR *ad libitum* and were fed up to 3 kg of concentrate per day in the AMS during milking. Silage and concentrate samples were collected every week. PMR samples were pooled over the course of the study to obtain the average. PMR was composed of a mean (± standard deviation) of 6.51 ± 0.04 MJ/kg dry matter, 35.5 ± 6.6% wheat and mineral mix, 28.7 ± 1.5% grass-clover silage, 26.8 ± 1.1% corn silage, 6.9 ± 6.0% barley, 0.6 ± 2.0% horse beans, and 0.5 ± 0.5% spring barley straw. The concentrate contained a mean of 18.2% crude protein and 10.2% crude fiber.

The number of visits and the duration of each visit to a feed bin were recorded using the automatized feeding bins. Individual cows were identified *via* a transponder attached to the ear. To calculate the daily eating time (min/d), the duration of each visit to a feeder (recorded by the Insentec Roughage Intake Control system) was summed over a day. Time intervals between visits were calculated for each cow from the stop time of the previous visit to the start time of the next visit. To determine if an interval was a part of a meal, we estimated a minimum interbout interval as follows. Time intervals measured in seconds were put in 1-min bins for the whole experimental period. Then, the average bin frequency was plotted against minutes. The x-axis was log-transformed to delineate the breakpoint clearly for this curve and, consequently, the threshold for meals (i.e., minimum interbout interval). The minimum interbout interval criterion was set at the breakpoint of 3 min, and time intervals shorter than this were deleted.

### Data handling

To investigate the effect of lameness and parity on feeding behavior, recordings obtained from an average of 59 individual cows per day were analyzed using SAS 9.4 (SAS Institute Inc., Cary, NC, USA). Due to data cleaning, however, data from six Jerseys cows (3 at first parity and 3 at a higher parity) were excluded from the analyses, as < 14 days of records were available within a parity. Additionally, data from 30 cows (14 at first parity, 16 at higher parities) were excluded from the analyses, as they had < 5 locomotion scorings within lactation. Additionally, data collected during the first 14 days of lactation were not included in the analyses. Similarly, any measurements exceeding 252 days (= 35 weeks) from calving were omitted from the analyses to exclude the effects of special handling of cows at the end of lactation. In addition, during a period of autumn of 2018, many cows were enrolled in other experiments and, therefore, fed differently. This led to exclusion of 63 dates to keep the numbers of cows similar across days. For the analyses, parity was dichotomized into first and second or higher lactation (primiparous and multiparous). Some cows who have more than one lactation were included.

After exclusions, data from 419 calendar dates from a total of 99 individual Jersey cows (contributing a mean of 235 days, range 70–390) remained available for the analyses. Cows could be lame and non-lame at different times during lactation. The total number of non-lame Jersey cows at first and later parities was 46, 71 and 20, and 51, respectively, were lame for at least one lactation week within the study period.

### Statistical analysis

The effects of lameness and parity, as well as their interaction, on eating time per visit, eating time per day, and between-meal intervals were analyzed by the linear mixed-effects models using the MIXED procedure in SAS. The GLIMMIX procedure with Gamma distribution and log link function was used to analyze the number of visits per day.

For the analyses, weekly averages of daily recordings were calculated and lameness status was assigned for this period as either lame or non-lame: To account for consecutive locomotion scorings, an average lameness score (ALS) was calculated for each cow and week. The LS was carried forward and placed on each day. Then, a backward moving average over 7 days was calculated using the EXPAND procedure in SAS:


(1) $$\begin{array}{l} Y_{kt}\;=\;\frac{1}{m}\left(\overset{m}{\underset{j=1}{\sum }}x_{k\left(t-m+j\right)}\right), \end{array}$$

where *Y*_*kt*_ is the ALS, *x*_*kt*_ is the LS at lactation day *t* = 15, …, 252 for cow *k* = 1, …, *n*, and *m* = 7 is the number of days to include in the time window.

**Figure 2:**
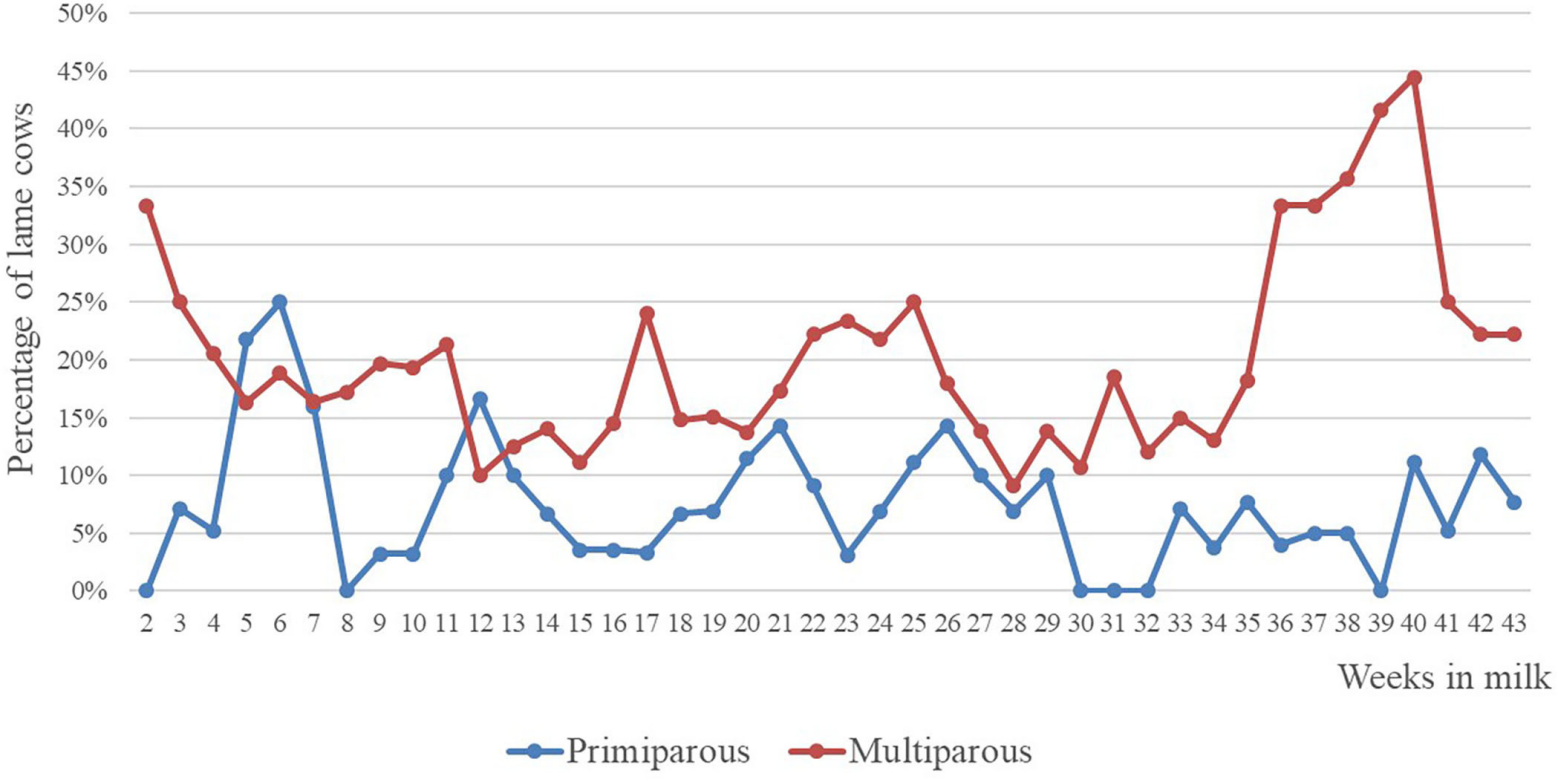
Percentage of cows assigned lame by parity and lactation week.

The daily ALS was averaged over weeks, and cows with a weekly average ALS of ≥ 2.5 were assigned as lame for this week. The percentage of animals-assigned lame among weeks in milk is shown in Figure 2.

Time (week in milk), parity (primiparous, multiparous), and lameness (lame, non-lame) and their two-way interactions were included as explanatory variables (fixed effects). Non-significant variables (*p* > 0.05) were eliminated from the model by backward elimination, resulting in different final models. However, the main effects of parity and lameness were always kept to not remove the relevant information. The final models are shown in the Supplementary File, none of which included the two-way interaction between parity and lameness.

Cow within parity was considered the experimental unit, and a continuous-time first-order autoregressive covariance structure was applied to account for repeated measures over weeks. Distributional assumptions and homogeneity of variances were examined by the graphical analysis of residuals for each model. Weekly averages of eating time per visit and per day and between-meal intervals were log-transformed (natural logarithm) to fulfill the assumption of normally distributed residuals.

For clarity, the results are reported on the original scale as exponentially back-transformed least squares means with 95% confidence intervals. The *p*-values from the Type 3 test for fixed effects were considered statistically significant when the *p*-value is ≤ 0.05. Further descriptions of the statistical analyses can be found in the online Supplementary File.

## Results

**Figure 3:**
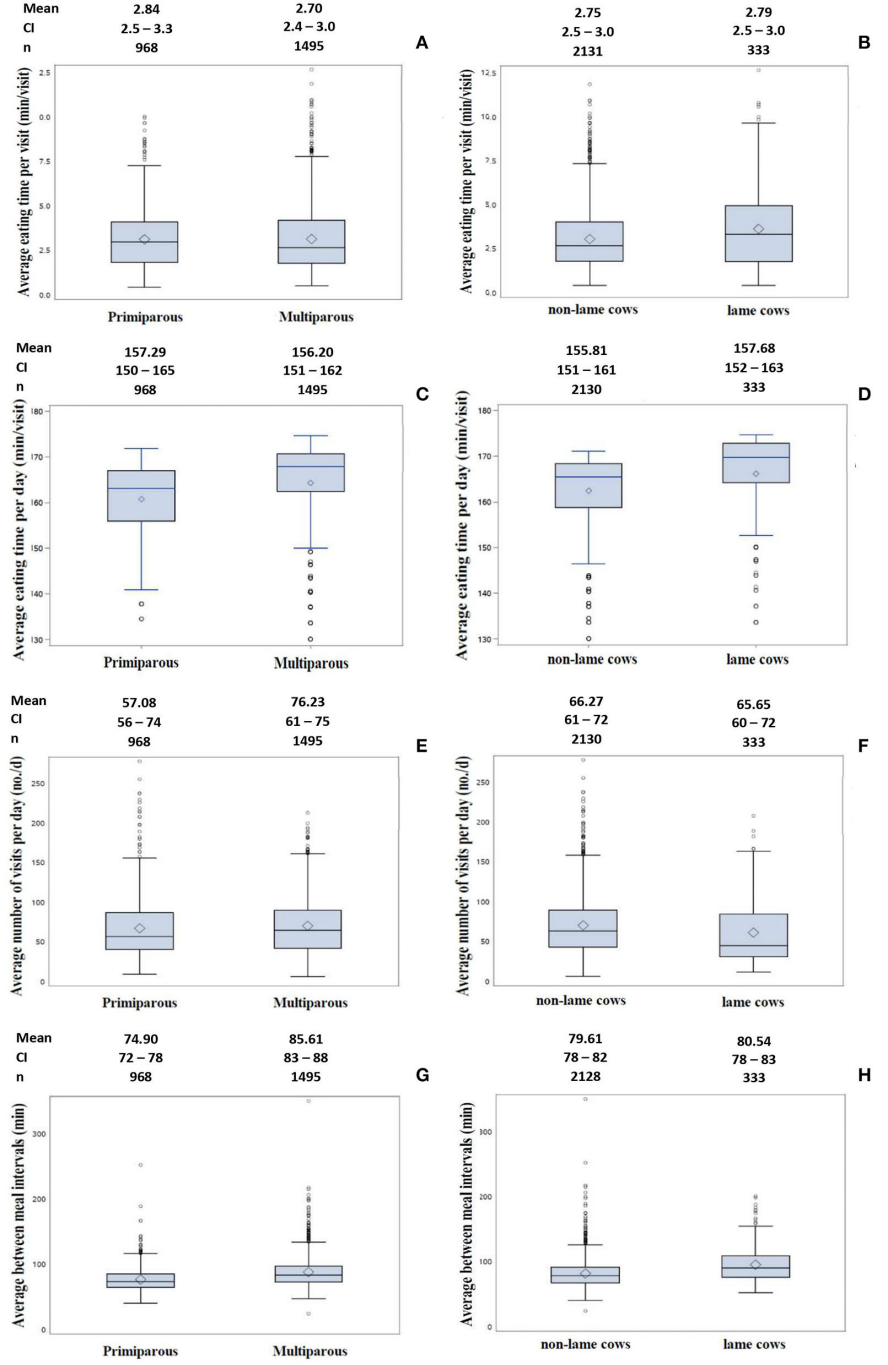
Distribution of eating time per visit, average number of visits per day, and between-meal intervals by parity **(A,C,E,G)** and lameness **(B,D,F,H)**, with the diamond inside the box indicating the mean value. The lower boundary of the box indicates the 25th percentile, the line inside the box is the median, and the upper boundary of the box INDICATES the 75th percentile. The whiskers represent the 10th and 90th percentiles, respectively. The bullet points are outliers, which are below or above the whiskers. The number of included measures as well as the back-transformed least squares mean with a 95% confidence interval is given above each boxplot.

**Figure 4:**
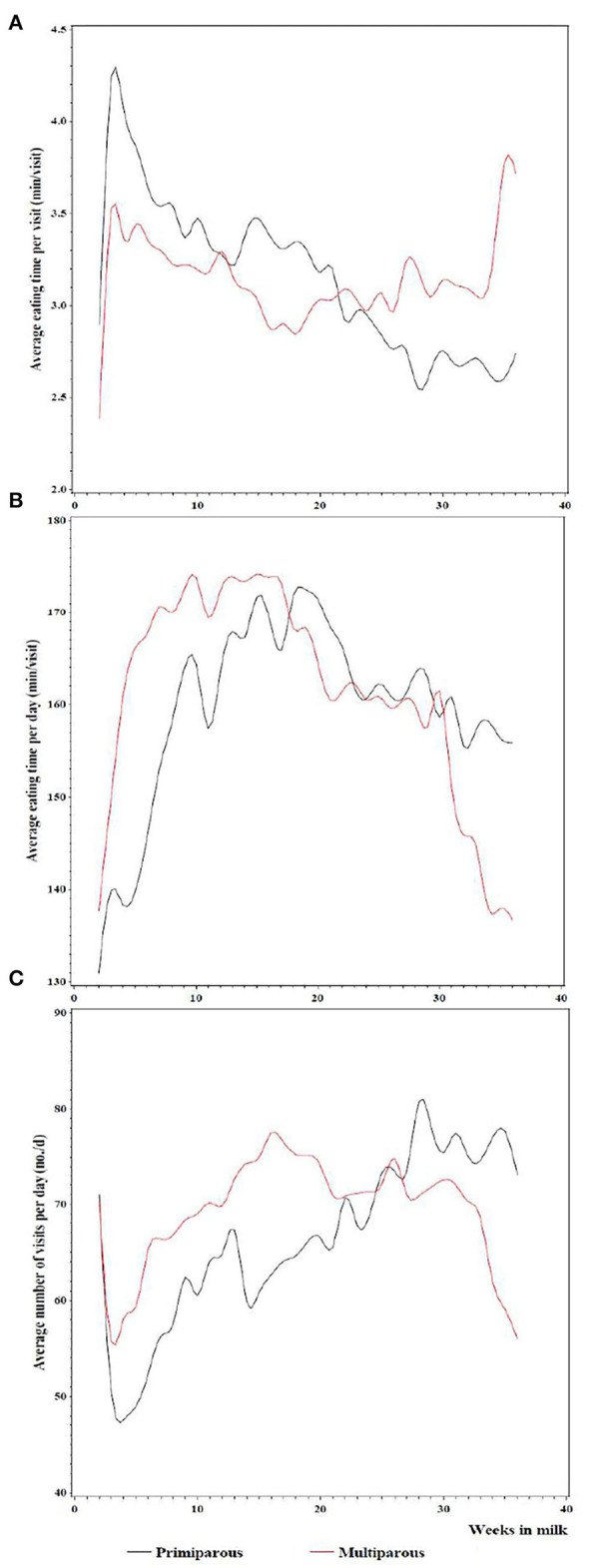
Average eating time per visit **(A)**, per day **(B)**, and the average number of visits per day **(C)** *vs*. weeks in milk for primiparous and multiparous Jersey cows. Daily records were averaged for each week in milk and each animal, and smoothed curves were drawn through the scatter points against weeks in milk.

There was no significant difference between parities for eating time per visit (Figure 3A). Primiparous cows spend 2.84 min per visit compared to 2.70 min per visit for older cows (*p* = 0.6090). Eating time per visit changed throughout lactation (*p* = 0.0065) with similar trends between parities. Visual inspection showed that the eating time per visit decreased over the first 25 weeks and subsequently remained constant (Figure 4A). Eating time per visit did not differ between lame and non-lame cows (*p* = 0.1504) who were eating for 2.79 and 2.75 min per visit, respectively (Figure 3B).

Parity influenced the daily eating time (*p* = 0.0090). We found that older Jersey cows spent 156.20 min per day eating compared to 157.29 for primiparous cows (Figure 3C). Additionally, eating time per day changed throughout lactation differing with respect to parity (*p* = 0.0007; Figure 4B). Until the 10th week of lactation, the daily eating time of multiparous cows increased sharply and decreased again from week 16, after staying relatively constant in between. Primiparous cows exhibited a moderate increase in the daily duration of eating until the 20th week, before slowly declining toward the end of lactation.

The effect of lameness on eating time per day was not significant (*p* = 0.2276). Lame cows spent 157.68 min per day eating while non-lame ate for 155.81 min per day (Figure 3D).

Figure 3E shows the distribution of an average number of visits per day by parity. With 57.08 visits per day, primiparous cows visited the feeder significantly fewer times (*p* = 0.0267) than multiparous cows having 76.23 visits per day. Week in lactation affected the number of visits significantly with the first order coefficient differing between parities (interaction *p* = 0.0117). The number of visits per day of primiparous cows increased steadily until the 29th week in lactation, whereas the number of visits of multiparous cows increased over the first 17 weeks of lactation and then decreased (Figure 4C). The effect of lameness on the number of visits per day was not significant (*p* = 0.4735). Lame cows had 65.65 visits per day while non-lame cows had 66.27 visits per day (Figure 3F).

The minimum between-meal interval was 10.71 min longer (*p* < 0.0001) for multiparous cows (85.61 min) compared to primiparous cows, spending at least 74.90 min between two meals, which can consist of multiple visits (Figure 3G). Time intervals between meals were not significantly different (*p* = 0.2799) for lame cows (80.54 min) compared to non-lame cows (79.61 min) with the distribution between meal intervals presented in Figure 3H.

## Discussion

### Lameness distribution

A wide variety of lameness definitions and different scoring systems that range from dichotomous scores of lame and non-lame up to the nine-point scales make it difficult to compare studies of lameness (3). Within the same scoring system, the lameness prevalence found in the present study is comparable to a previous Danish study using 1,340 cows from 42 dairy herds, of which 508 (38%), 437 (33%), 232 (17%), 121 (9%), and 42 (3%) have been scored with LS 1, 2, 3, 4, and 5, respectively. However, their study included Jersey and Holstein cows. A study of four farms with a total of 959 LS from 348 Danish Holstein cows had a slightly higher lameness prevalence with a score of 1, 2, 3, 4, and 5, respectively, for 325 (34%), 276 (29%), 194 (20%), 141 (15%), and 23 (2%) cows (17).

To our knowledge, there is no study with pure Jersey cows within Denmark using the described scoring system.

### Duration of eating time per visit and per day

We hypothesized that the eating time per visit would be longer for lame cows than for non-lame cows. Interestingly, we found no differences in the time spent eating per visit between non-lame and lame cows. Our findings are in agreement with previous studies (18, 23) which showed no differences between lame and non-lame cows for eating time per visit.

With the cows being fed *ad libitum*, it is possible that lame cows were able to use other times of the day to avoid competition at the feeders and fulfill their needs without changing the duration of their visits. We suggest further research with a sufficiently large dataset investigating if lame cows feed at different times of the day or if differences in other feeding variables such as the feeding rate occur. Contrary to our results, previous studies found that lame cows fed fewer and shorter meals (16, 24). It is possible that lame cows try to reduce the frequency of activities that tend to be painful and, therefore, feed in fewer but longer visits. This is in agreement with the results of Thorup et al. (17), who found reduced activity in lame dairy cows. The number of visits between lame and non-lame cows was not significantly different.

Our expectation of increasing eating time per visit with parity was not confirmed. We observed no significant differences between parities. Val-Laillet et al. (25) assumed that the motivation or persistence of the animal plays a role in competitive success to gain access to feeders. If primiparous cows are more motivated to feed due to their high energy requirements for growth and milk production, they would be displaced less often by others and, therefore, have longer visit times. Further, older cows having a greater eating rate and spending more time lying and ruminating and, thus, less time eating (14) might explain this phenomenon. In our study, primiparous cows spent more time eating per day compared to multiparous cows. This result is in contrast to our observation for feeder visits per day; thus, older cows visited the feeder more often but spent less time eating per day but not per visit. This finding was unexpected, as dry matter intake, eating time, and feeder visits are correlated (26). Further, it should be noted that the difference, even though statistically significant, numerically is small and, therefore, might be biologically irrelevant. Some studies support the assumption of eating time increasing with parity (27, 28), whereas others found that younger cows spend more time eating than older cows (29, 30). These differences between studies might be attributed to different experimental conditions, such as feed composition or forage ratios affecting eating behavior (31).

### Number of visits

Contrary to our hypothesis, we found no statistically significant difference in feeder visits between lame and non-lame Jersey cows.

The rather high stocking density in our study might have affected the number of visits to the feeder, increasing competition and causing animals to be more frequently displaced from feeders and, therefore, limiting differences between them. Further, it is well documented that restricting access to feed increases the frequency of displacement, especially for subordinate cows (32, 33). Therefore, we had expected that, with increasing lameness severity, lame cows might reduce the number of visits to limit confrontations and likely painful movements such as walking, getting up, and lying down. We suggest additional research with larger samples to further test the hypothesis that lameness impairs the number of visits per day in Jersey cows.

Jersey cows showed the pattern of multiparous cows visiting the feeder significantly more times compared to primiparous cows disagreeing with studies with Holstein cows (27, 34, 35). Further, it contradicts previous studies on Jerseys, which found no difference between parities (36) or reported primiparous cows to be having a higher frequency of feeder visits (35). Possibly due to their lower body weight, primiparous cows inherit a lower rank within the herd, forcing them to visit the feeder at less bustling times (37, 38), while, at the same time, first parity cows increased the time feeding per day and with that possibly compensating for the lesser visits. Further, older cows tend to lie down for longer and have higher milk yields and body weights (18, 39), increasing their motivation for feeding but shortening the time available. Thus, multiparous cows might optimize their active time by visiting the feeders more often within a meal, but having fewer meals per day and, therefore, longer intervals between meals.

The increase in feeder visits during early lactation in our study likely compensated for an increase in energy demand during early to peak lactation (26, 40).

### Between meal intervals

An increase in lameness severity often leads to decreasing time devoted to eating (24). As most movements like getting up or lying down are painful for lame cows, affected cows are thought to limit their feeding bouts (3, 41) and, therefore, the time between meals would increase. Additionally, fewer visits to the feeder are likely to result in fewer confrontations with herd mates. Our findings do not support this, with intervals between meals not being different for lame cows. This is similar to findings by Blackie and Maclaurin (42), which found no statistically significant differences in the lying behavior of lame and non-lame zero-grazed Jersey cows. Together with our findings, this may raise concerns about the suitability of behavioral reactions to detect lameness occurrences in Jersey cows. It also highlights that comparing breed differences is an important issue for future research.

Compared to multiparous cows, primiparous Jersey cows had significantly shorter between-meal intervals, supporting our hypothesis. The between meal intervals increased with parity. This might be explained by older cows spending more time ruminating and thus having fewer meals over a day (27). Our findings of longer between meal intervals are in agreement with findings of higher parity cows visiting the feeder significantly fewer times compared to younger cows (34, 35), which consequently increases the time between visits. Yet, in our study, we found multiparous cows having more feeder visits compared to first parity cows. The reason for this result is not clear. It is possible that older Jersey cows will visit the feeders more often within a meal, while having fewer meals over a day and, therefore, longer between-meal intervals.

## Conclusion

This study showed that, contrary to previous research in other breeds, no differences were found in the eating time per visit, the daily eating time, and feeding frequency for lame and non-lame Jersey cows.

Although the amount of data was limited as data from only one herd were used, this study provides a first indication that Jersey cows could react differently to lameness compared to other breeds, namely the predominant Holstein breed, and that feeding parameters might not be used as an early indicator of the onset of lameness in Jersey cows. However, further studies are needed to confirm the findings.

## Data availability statement

The datasets presented in this article are not readily available because all data belongs to the Aarhus University. Requests to access the datasets should be directed to Department of Animal Science Aarhus University anis@au.dk.

## Ethics statement

Ethical review and approval was not required for the animal study because the animals have not been directly affected according to European and Danish regulations, and current guidelines for the ethical use of animals in research.

## Author contributions

SG and LF analyzed the data. SG, CL, LF, and PT interpreted data. SG drafted the initial manuscript. CL, LF, and PT provided critical revisions and improvements. All authors contributed to the article and approved the submitted version.

## Funding

The project was financially supported by the Federal Ministry of Education and Research (Berlin, Germany, programme FHprofUnt 2018). We acknowledge support for the Article Processing Charge from the Deutsche Forschungsgemeinschaft (DFG, German Research Foundation, 491232355) and the Open Access Publication Fund of the Hochschule Neubrandenburg (Neubrandenburg University of Applied Sciences).

## Conflict of interest

The authors declare that the research was conducted in the absence of any commercial or financial relationships that could be construed as a potential conflict of interest.

## Publisher's note

All claims expressed in this article are solely those of the authors and do not necessarily represent those of their affiliated organizations, or those of the publisher, the editors and the reviewers. Any product that may be evaluated in this article, or claim that may be made by its manufacturer, is not guaranteed or endorsed by the publisher.
