# Supplementary material for: Effect of lameness on feeding behavior of zero grazed Jersey dairy cows
Source: Front Vet Sci. 2022 Sep 20;9:980238. doi: 10.3389/fvets.2022.980238 (PMC9530783; doi:10.3389/fvets.2022.980238)
Supplement: Supplementary file 1 [file Data_Sheet_1.PDF]

Supplementary Material

1 Supplementary statistics

To analyze the effect of lameness and parity on eating time per visit, the following model was used:

$$log(Y_{ijkl}) = L_i + P_j + w_{ijkl} + \varepsilon_{ijklm} \qquad (Model\ 1)$$

Here Y_{ijkl} is the response variable (eating time per visit), L_i is effect of lameness (i = lame, non-lame), P_j is effect of parity (j = 1, 2+), w_{ijkl} is the effect of weeks in lactation, and $\varepsilon_{ijkl} \sim N(0, \sigma^2)$ are the random residuals where *l* index the repeated measures over weeks for cow $k = 1, \dots, n_{ij}$. Correlation among observations from the same cow within parity was modelled using the SP(POW) covariance structure in SAS (corresponding to a continuous-time AR(1) correlation structure), using week in lactation to specify difference between time points, i.e. covariances of the form $cov(Y_{ijkl}, Y_{ijkm}) = \sigma^2 \rho^{|w_{ijkl} - w_{ijkm}|}$, where ρ is the correlation coefficient.

To analyze the effect of lameness and parity on between meal intervals, Model 1 was used although excluding the covariate for weeks in lactation as it was not significant (P > 0.05).

To analyze the effect of lameness and parity on eating time per day, a second order polynomial was used for weeks in milk to better fit nonlinear changes during lactation. This resulted in the following model:

$$\log(Y_{ijkl}) = L_i + P_j + (\beta_{P_j})w_{ijkl} + (\gamma_{P_j})w_{ijkl}^2 + \varepsilon_{ijkl} \qquad (Model\ 2)$$

In addition to the parameters described for Model 1, β_{P_j} is the parity-specific slope parameter for weeks in lactation, w_{ijkl} and γ_{P_j} is the parity-specific parameter for weeks in lactation squared, w_{ijkl}^2.

The effect of lameness and parity on average number of visits per day (averaged within week) was analyzed by a generalized linear mixed effects model with Gamma distribution and log link function. The linear function of means on the link scale was:

$$\text{mean}(\log(Y_{ijkl})) = L_i + P_j + (\beta_{P_j})w_{ijkl} + (\gamma_{P_j})w_{ijkl}^2 \qquad (Model\ 3)$$

The parameters are described for Model 2.
